# How Microbes Defend Themselves From Incoming Hydrogen Peroxide

**DOI:** 10.3389/fimmu.2021.667343

**Published:** 2021-04-27

**Authors:** Ananya Sen, James A. Imlay

**Affiliations:** Department of Microbiology, University of Illinois at Urbana-Champaign, Urbana, IL, United States

**Keywords:** reactive oxygen species, OxyR regulator, peroxide sensing repressor (PerR), Yap1p, nitric oxide

## Abstract

Microbes rely upon iron as a cofactor for many enzymes in their central metabolic processes. The reactive oxygen species (ROS) superoxide and hydrogen peroxide react rapidly with iron, and inside cells they can generate both enzyme and DNA damage. ROS are formed in some bacterial habitats by abiotic processes. The vulnerability of bacteria to ROS is also apparently exploited by ROS-generating host defense systems and bacterial competitors. Phagocyte-derived ${\text{O}}_{2}^{-}$ can toxify captured bacteria by damaging unidentified biomolecules on the cell surface; it is unclear whether phagocytic H_2_O_2_, which can penetrate into the cell interior, also plays a role in suppressing bacterial invasion. Both pathogenic and free-living microbes activate defensive strategies to defend themselves against incoming H_2_O_2_. Most bacteria sense the H_2_O_2_ *via* OxyR or PerR transcription factors, whereas yeast uses the Grx3/Yap1 system. In general these regulators induce enzymes that reduce cytoplasmic H_2_O_2_ concentrations, decrease the intracellular iron pools, and repair the H_2_O_2_-mediated damage. However, individual organisms have tailored these transcription factors and their regulons to suit their particular environmental niches. Some bacteria even contain both OxyR and PerR, raising the question as to why they need both systems. In lab experiments these regulators can also respond to nitric oxide and disulfide stress, although it is unclear whether the responses are physiologically relevant. The next step is to extend these studies to natural environments, so that we can better understand the circumstances in which these systems act. In particular, it is important to probe the role they may play in enabling host infection by microbial pathogens.

## The Threat Posed by Oxygen

Life evolved 3.8 billion years ago in an anoxic world. The biochemical pathways of these primordial organisms were based upon iron-cofactored enzymes, as this transition metal is adept at both redox and ligand-exchange processes. One billion years later, the appearance of photosystem II began the release of diatomic oxygen into the atmosphere. The oxygen levels in the atmosphere remained relatively low for another billion years, because photosynthetically generated O_2_ was quickly consumed through its chemical reduction by environmental ferrous iron and sulfide. Only later, once these reductants had been largely titrated, did oxygen accumulate to higher concentrations (1). However, when it did, O_2_ created an environment that was—and remains—incompatible with extant organisms, which have inherited their iron-centric metabolic plans from their anoxic forbears.

In part the problem is that molecular oxygen oxidizes ferrous iron to insoluble ferric hydroxide precipitates, making it difficult for cells to acquire enough iron to charge their enzymes. That problem has been substantially ameliorated by the evolution of a variety of iron-import tactics (2). However, in addition, oxygen is kinetically active as a univalent oxidant (3). It disrupts the metabolism of anaerobic organisms by oxidizing their low-potential metal cofactors, thereby inactivating key enzymes, and by adducting the radical-based enzymes that play specialized roles in metabolism. Contemporary anaerobes have not solved this problem: In order to optimize their anaerobic growth, they continue to rely upon enzymes that are directly disrupted by oxygen, and so these microbes are constrained to anoxic niches (4).

**Figure 1:**
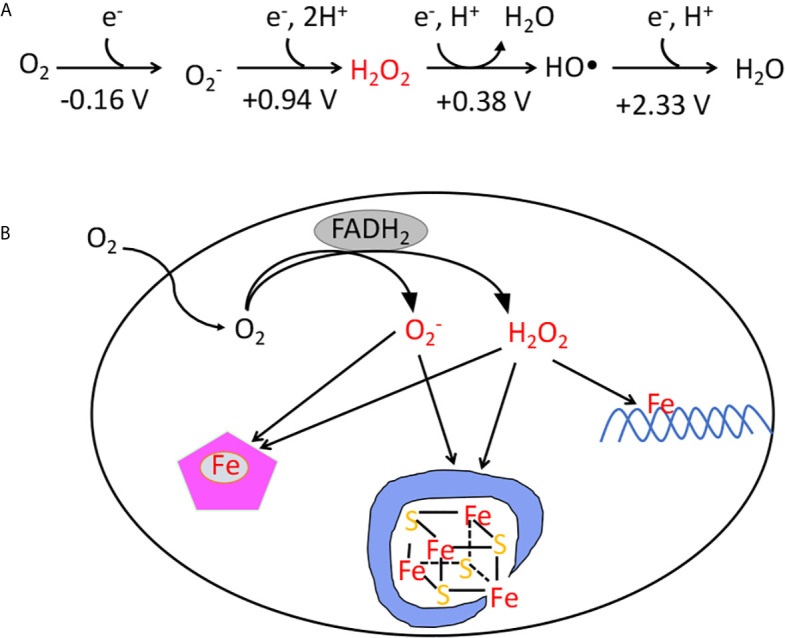
**(A)** The reduction potential of oxygen and reactive oxygen species. The standard reduction potentials (pH 7) indicate that unlike O_2_, superoxide, hydrogen peroxide, and hydroxyl radicals are potent univalent oxidants. The standard concentration of oxygen is regarded as 1 M. **(B)** The classes of damage caused by intracellular ${\text{O}}_{2}^{-}$ and H_2_O_2_. The transfer of electrons from redox enzymes to oxygen generates superoxide and hydrogen peroxide. Both species can oxidize the solvent-exposed iron centers of mononuclear iron enzymes and [4Fe-4S] dehydratases. Additionally, H_2_O_2_ directly reacts with the intracellular iron pool, which is loosely associated with biomolecules, including DNA. The reaction generates hydroxyl radicals, which can damage DNA.

In contrast, organisms that committed to life in oxic environments were able to dispense with low-potential catalytic strategies, and they employ enzymes that molecular oxygen does not damage at an important rate. Yet aerobes have residual problems with oxygen. Molecular oxygen is a passable univalent oxidant, and inside cells it adventitiously steals electrons from the cofactors of redox-active enzymes (5, 6). The transfer of a single electron results in the formation of superoxide (**Figure 1A**); the transfer of two electrons results in the formation of hydrogen peroxide. Both these species are more-potent univalent oxidants than molecular oxygen itself, and if left unchecked they can oxidize the exposed iron cofactors of enzymes that are found throughout metabolism. Further, a secondary reaction between hydrogen peroxide and cellular iron pools creates hydroxyl radicals (**Figure 1**), which are extremely potent species that can directly oxidize all cellular biomolecules (7). This vulnerability to partially reduced oxygen species (ROS) is universal among contemporary organisms. This review specifically aims to describe the strategies that are used by organisms to defray the toxicity of hydrogen peroxide—and to highlight the circumstances in which these defenses may not be adequate.

## Reactive Oxygen Species are Continuously Formed Inside Oxic Cells

The significance of hydrogen peroxide (H_2_O_2_) was first suggested by the discovery in 1900 of an enzyme devoted to degrading it: catalase (8). The discoverer, Oscar Loew, noted that it is found in virtually all tissues, and he made the inference that H_2_O_2_ was likely a by-product of metabolism that, if not removed, must be toxic to cells. Seventy years later Joe McCord and Irwin Fridovich chanced upon an enzyme that degrades superoxide (${\text{O}}_{2}^{-}$) (9). Subsequent work has extended the cohort of scavenging enzymes to include peroxidases and superoxide reductases, and it has confirmed that virtually no organism lacks the ability to degrade H_2_O_2_ and ${\text{O}}_{2}^{-}$.

**Figure 2:**
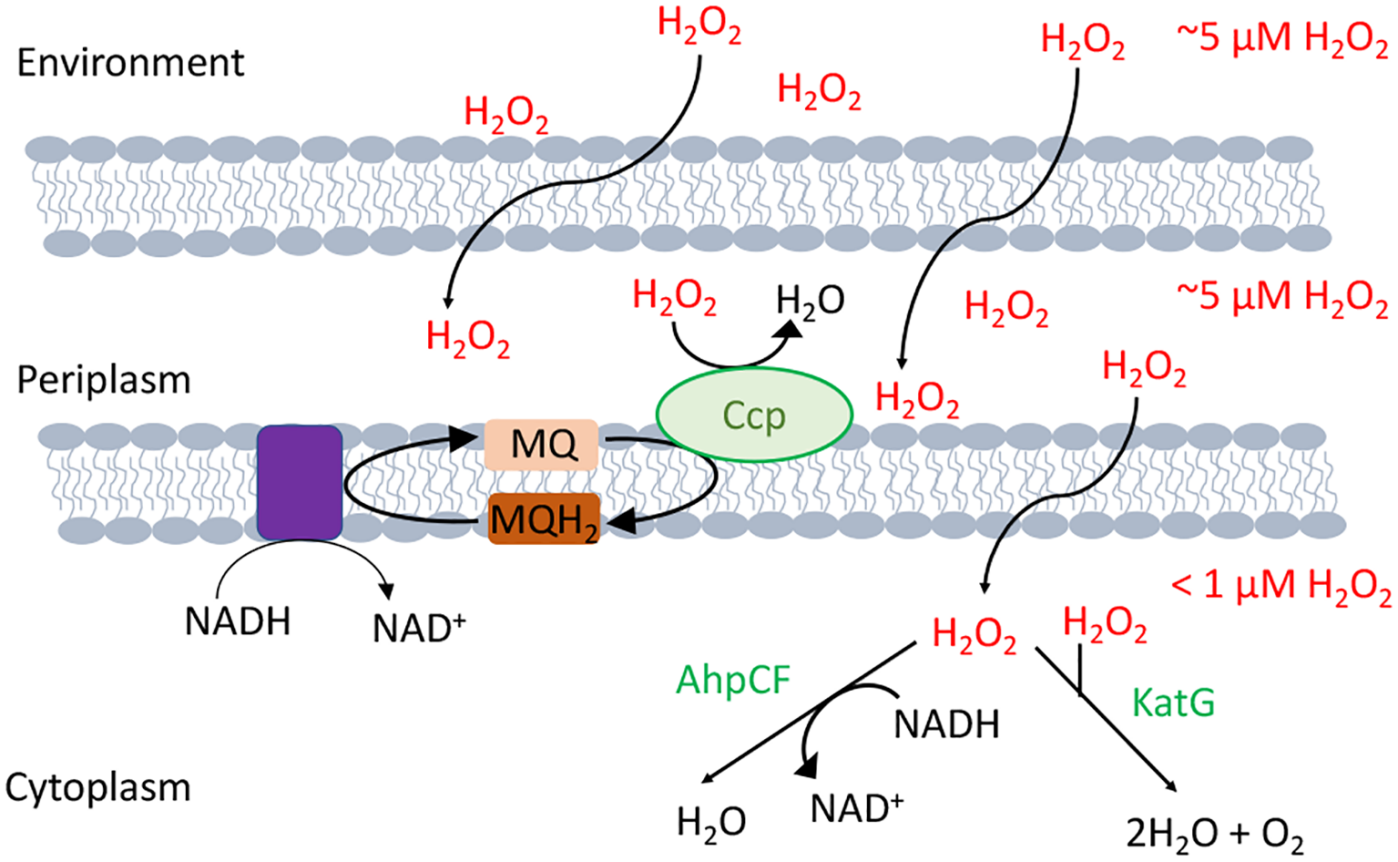
H_2_O_2_-scavenging enzymes in *E. coli*. Environmental H_2_O_2_ gradually diffuses into the cytoplasm, where it is degraded by NADH peroxidase (AhpCF) and catalase (KatG). Both are induced by OxyR. Cytoplasmic H_2_O_2_ is therefore substantially lower in concentration than is extracellular H_2_O_2_. Under hypoxic conditions OxyR also induces the periplasmic cytochrome c peroxidase (Ccp), which allows the respiratory chain to employ H_2_O_2_ as a terminal oxidant. Because H_2_O_2_ rapidly crosses through OM porins, and Ccp activity is moderate, the periplasmic H_2_O_2_ concentration is likely equivalent to that outside the cell.

The model bacterium *Escherichia coli* contains two superoxide dismutases in its cytoplasm and one in its periplasm. Its cytoplasm also features both an NADH peroxidase (AhpCF) and two catalases (10) (**Figure 2**). Interestingly, some of these enzymes take advantage of the fact that iron can react with ${\text{O}}_{2}^{-}$ and H_2_O_2_: The original superoxide dismutase was likely an iron-dependent SOD, and most catalases use heme to degrade H_2_O_2_. Looking more broadly, similar scavenging systems are distributed through all biological kingdoms and in most cellular compartments, including the mitochondria, peroxisomes, and cytoplasm of eukarya.

The importance of these enzymes was revealed by genetic studies of *E. coli*: Mutants that lack cytoplasmic SODs, or that lack Ahp and catalases, were found to be unable to grow under oxic conditions in a standard glucose medium (10, 11). The observation confirmed Loew’s postulate, proving both that these species are generated internally and that if not scavenged they will cripple cellular metabolism. Direct observation of ${\text{O}}_{2}^{-}$ production is not possible, but mutants that cannot degrade H_2_O_2_ release it into growth media at a rate that connotes an internal production rate of 10 µM/sec (12). Measurements show that as little as 0.5 µM intracellular H_2_O_2_ is sufficient to poison select biosynthetic pathways (13–15). The titers and kinetics of the scavenging enzymes are sufficient to suppress internal concentrations to about 50 nM—low enough to enable metabolism to operate without bottlenecks (12).

The orbital structure of O_2_ restricts it to accepting a single electron at a time, and its reduction potential (- 0.16 V) is too modest for it to directly oxidize most biomolecules (3). However, O_2_ can accept electrons from electron donors such as metal centers, flavins, and quinones. These are all prominent electron carriers in the *E. coli* respiratory chain, yet the rate at which cells produce endogenous H_2_O_2_ did not substantially diminish in mutants that lacked the respiratory enzymes, suggesting that in this bacterium ${\text{O}}_{2}^{-}$ and H_2_O_2_ are primarily produced by the accidental autooxidation of non-respiratory flavoproteins (5, 6) (**Figure 1**). These proteins are found throughout metabolism, and many, including glutathione reductase, lipoamide dehydrogenase, and glutamate synthase, have been shown to release ROS *in vitro* (16–18). The small size of O_2_ prevents its exclusion from most active sites, and its collision with reduced flavins triggers the consecutive transfer of one or two electrons, generating ${\text{O}}_{2}^{-}$ or H_2_O_2_, respectively (19). The rate is naturally proportionate to collision frequency, meaning that ROS formation is more rapid in highly oxic environments. This math presumably underlies the observation that most organisms cannot tolerate oxygen levels that substantially exceed those of their natural habitat.

## The Classes of Damage Caused by Superoxide and Hydrogen Peroxide

The phenotypes of SOD mutants and of catalase/peroxidase mutants enabled investigators to track down the specific injuries that these ROS create. The mutants can grow at wild-type rates under anoxic conditions, but they require supplementation with aromatic and branched-chain amino acids if they are to grow in oxic media (11, 13–15, 20). They are also unable to use any carbon source, such as acetate, that required a fully functional TCA cycle (21, 22). Further scrutiny identified particular enzymes whose damage resulted in these defects.

**Figure 3:**
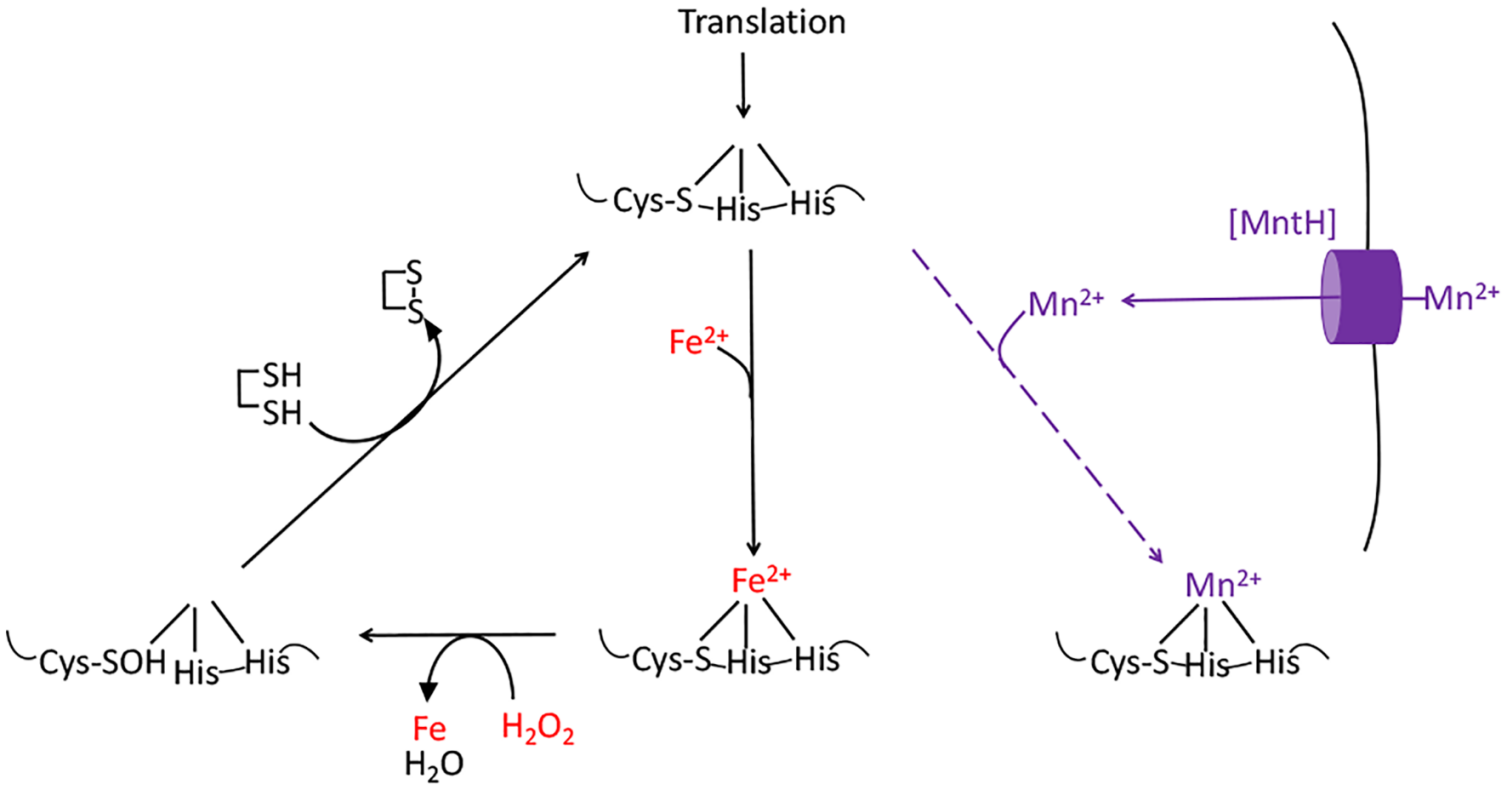
The damage caused to mononuclear iron proteins by hydrogen peroxide. H_2_O_2_ directly oxidizes the solvent-exposed Fe(II) cofactor, which then dissociates. The ferryl (FeO^2+^) species that is formed in this reaction can directly oxidize the polypeptide ligands to the iron atom, irreversibly inactivating the enzyme. However, if a cysteine residue coordinates the iron, it will quench the ferryl radical (as shown). The enzyme activity can then be restored by reduction of the cysteine sulfenate residue, probably by thioredoxins. OxyR induces the MntH manganese importer, allowing the proteins to be metallated with Mn(II), which provides activity and does not react with H_2_O_2_.

The aromatic biosynthesis defect derives from the ability of H_2_O_2_ to oxidize the Fe(II) cofactor of the first enzyme of the pathway, DAHP synthase (15). The resulting Fe(III) atom dissociates. The role of the iron atom is both to bind substrate and to stabilize an oxyanion intermediate in the catalytic cycle; therefore, the resultant apoenzyme is completely inactive and the pathway fails. Other mononuclear Fe(II) enzymes such as ribulose-5-phosphate 3-epimerase, peptide deformylase, threonine dehydrogenase, and cytosine deaminase are similarly damaged (13, 14). Notably, the reaction between Fe(II) and H_2_O_2_ also generates a hydroxyl radical. If the catalytic Fe(II) atom is coordinated by a cysteine side chain, the hydroxyl radical reacts immediately with this sacrificial residue, creating a sulfenic acid (**Figure 3**) (13). Cellular thioredoxins and glutaredoxins can reduce this moiety back to a native cysteine residue, thereby allowing Fe(II) to bind and the activity to be restored (13). In contrast, when H_2_O_2_ oxidizes the Fe(II) of enzymes that lack such a residue, the nascent hydroxyl radical oxidizes other active-site ligands, creating lesions that are irreversible. These reactions are one source of the protein carbonylation that can be detected in H_2_O_2_-stressed cells (13).

**Figure 4:**
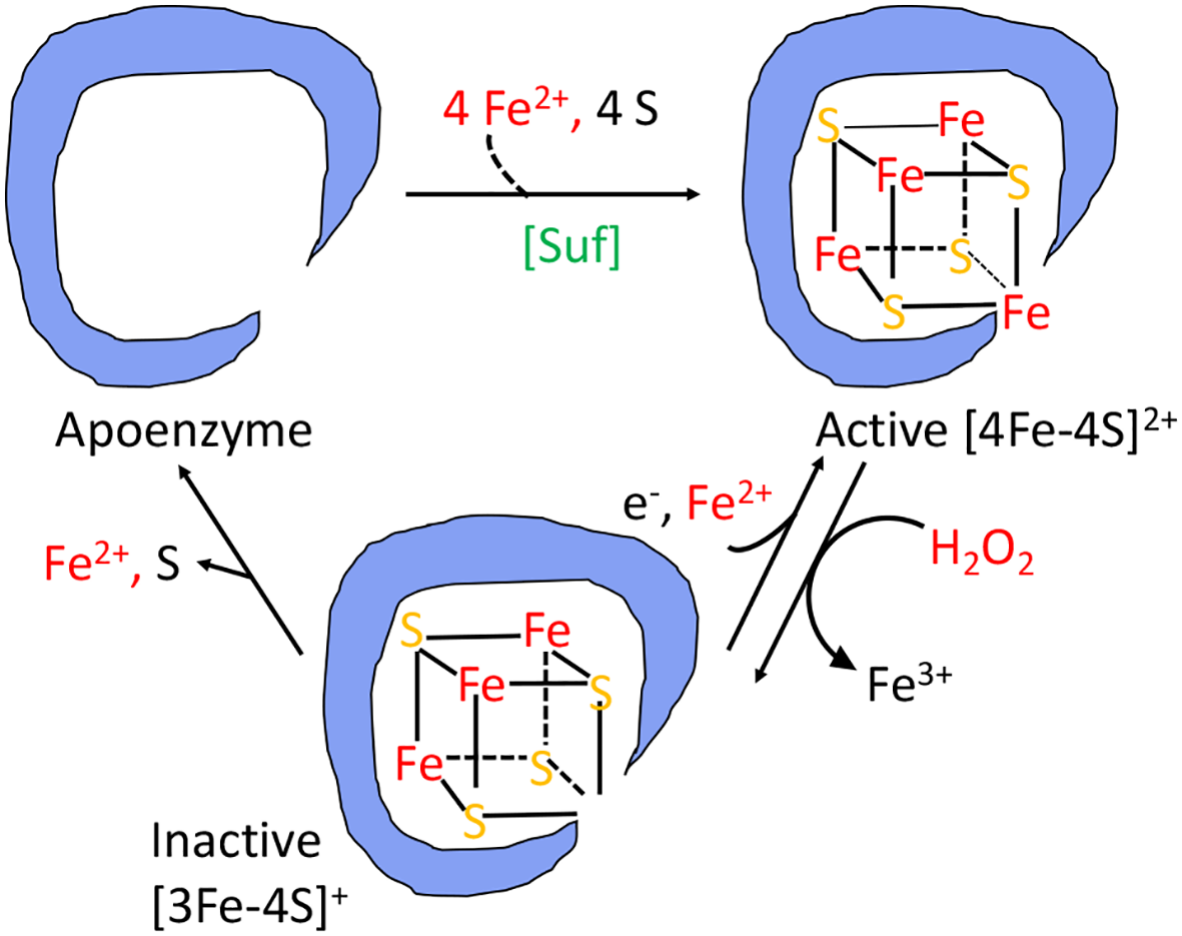
The damage caused to [4Fe-4S]^2+^ cluster enzymes by hydrogen peroxide. The catalytic Fe atom of the dehydratase enzyme reacts with H_2_O_2_ and dissociates, leaving behind an inactive [3Fe-4S]^+^ cluster. That cluster can be reactivated by reduction and remetallation. In some dehydratases the cluster completely disintegrates to form an apoenzyme. OxyR induces the Suf system to rebuild a functional holoenzyme.

The branched-chain biosynthetic defects as well as the TCA-cycle defects are caused by the oxidation of the [4Fe-4S] clusters of dehydratase enzymes (20, 22–26). A solvent-exposed iron atom of these clusters binds substrate directly, activating it for deprotonation and subsequently completing the dehydration by abstracting a hydroxide anion. But the same exposed iron atom can be oxidized by hydrogen peroxide to an unstable [4Fe-4S]^3+^ state (**Figure 4**); this valence is unstable, and the cluster quickly disintegrates into a [3Fe-4S]^+^ form that lacks the catalytic, solvent-exposed iron atom (24). Interestingly, the hydroxyl radical that is formed during this process pulls a second electron from the iron-sulfur cluster; thus, a hydroxide anion, rather than a hydroxyl radical, is released into the active site, and polypeptide oxidation is avoided. Cells continuously repair these damaged clusters, so that the steady-state activity of these enzymes reflects the balance between the oxidation and repair rates (21, 27, 28).

In addition to the metabolic defects described above, H_2_O_2_ can also react with the cytoplasmic pool of loose iron that is used to metallate nascent iron-dependent enzymes (**Figure 1B**). Iron is sticky, and this pool is thought to adhere to a wide variety of biomolecules, including the surface of nucleic acids (29). DNA thereby acts as a locus of hydroxyl radical production, and so DNA damage is a universal consequence of H_2_O_2_ stress (30–33). Some oxidative base lesions are mutagenic; others comprise replication blocks. All organisms therefore wield enzymes devoted to the excision or recombinational repair of oxidative lesions. Even though the level of endogenous H_2_O_2_ is well-controlled in scavenger-proficient *E. coli*, the rate of DNA oxidation remains high enough that mutants lacking these repair pathways cannot grow in oxic environments (34, 35).

Thus, organisms that dwell in oxic habitats can do so only because they have acquired an array of both scavenging and repair functions. The level of these enzymes is high and their synthesis is costly; accordingly, their titers have been calibrated to barely withstand the amount of stress commensurate with the oxygen level of the native environment (36, 37). This arrangement is successful under routine growth conditions. However, we shall see that it becomes inadequate if special circumstances elevate the production of ROS.

## The Problem of Exogenous Oxidative Stress

### Exogenous Sources of Superoxide

Both environmental photochemistry and chemical redox reactions generate ${\text{O}}_{2}^{-}$ (38), but the steady-state level of ${\text{O}}_{2}^{-}$ formed in this way is unlikely to be high enough to pose a risk for cells. Notably, the known targets of ${\text{O}}_{2}^{-}$ are iron enzymes that are cytoplasmic, and ${\text{O}}_{2}^{-}$ is a charged species that cannot cross membranes to get at them (39, 40). However, both microbes and higher organisms have evolved mechanisms by which they can use ${\text{O}}_{2}^{-}$ to poison unwanted competitors.

**Figure 5:**
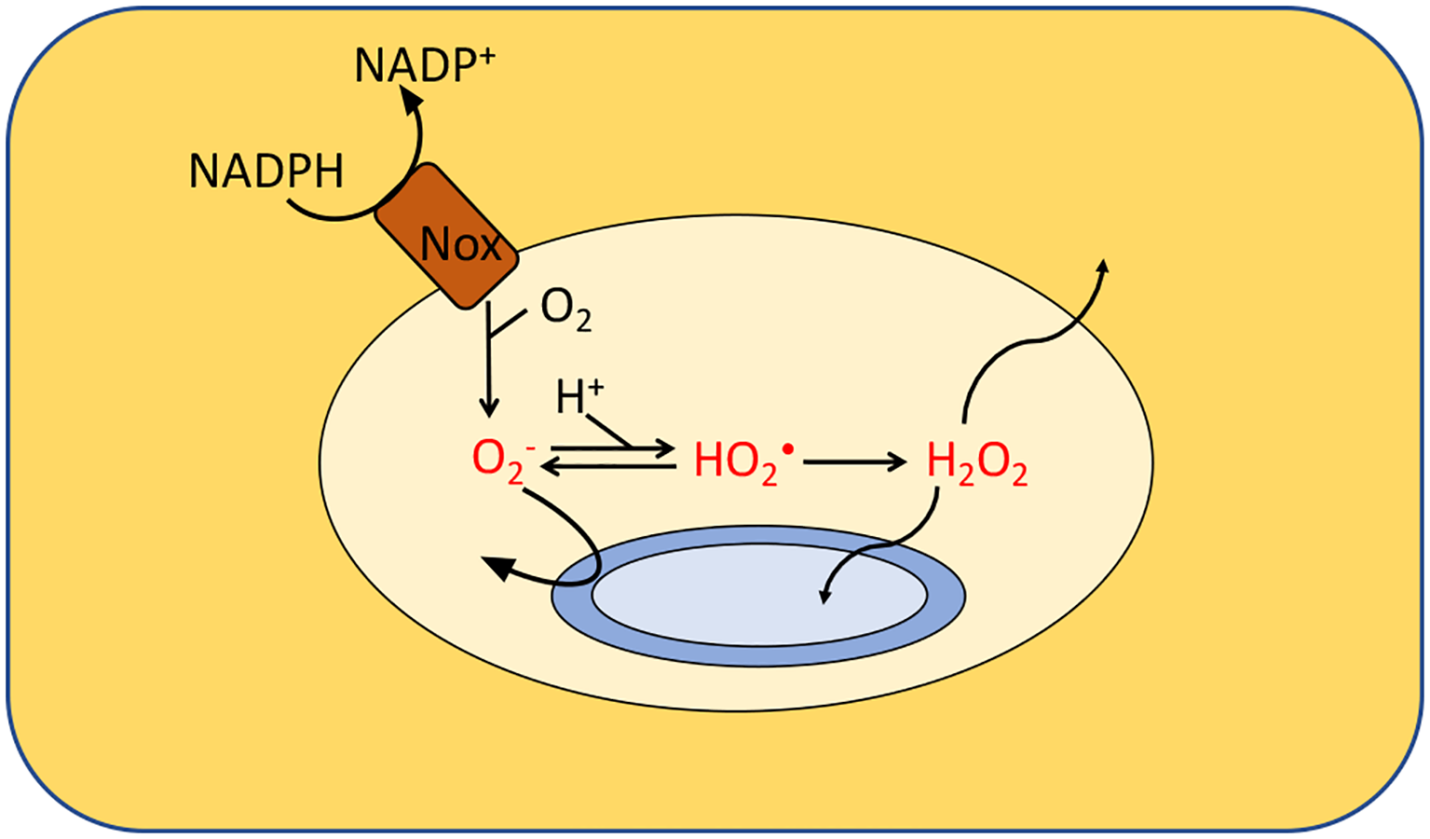
Formation of ROS by phagosomes. NADPH oxidase (Nox) generates superoxide which cannot penetrate the cytoplasmic membranes of the engulfed bacteria. It is believed that either superoxide or its protonated form injures extracytoplasmic targets. Additionally, membrane-permeable H_2_O_2_ is generated through dismutation. Calculations suggested that the levels of ${\text{O}}_{2}^{-}$, HO_2_• and H_2_O_2_ in isolated macrophages range from 10–50 μM, 0.1–4 μM and 1–4 μM, respectively, depending upon phagosomal pH. Modeling predicts a similar H_2_O_2_ concentration inside neutrophils. The H_2_O_2_ levels would rise, however, if it accumulates it the surrounding tissue.

Mammals, plants, and amoebae have all weaponized an NADPH oxidase to kill bacteria (41, 42). Mammalian phagocytes engulf microbial invaders and spray them with superoxide that is formed by an inducible NADPH oxidase (**Figure 5**). The importance of this enzyme is reflected by the observation that humans and mice that lack it are vulnerable to infections (43, 44). However, it is still unclear how the phagocytic ROS production inhibits microbial growth. The acidic environment inside the phagosome can partially protonate ${\text{O}}_{2}^{-}$, resulting in a neutral species that in principle can penetrate captive bacteria; however, *Salmonella enterica* mutants lacking the periplasmic superoxide dismutase are hypersensitive, suggesting that ${\text{O}}_{2}^{-}$ does not gain access into the cytosol and instead acts on a target on the cell surface or in the periplasm (45, 46). Because protonated HO_2_ is a better oxidant than ${\text{O}}_{2}^{-}$, it is possible that the acidity of the phagosome expands the range of biomolecules that superoxide can damage. The target has not yet been identified. A key difficulty is that *in vitro* systems have been unable to match the micromolar doses of superoxide (46, 47) that are sustained in the phagosome.

Bacteria also have found a way to impose ${\text{O}}_{2}^{-}$ stress upon competitors—and in this case the ${\text{O}}_{2}^{-}$ is aimed at the cytoplasm. A wide range of bacteria (and plants) secrete redox-cycling antibiotics (48–51). These are primarily soluble quinones and phenazines; they penetrate target cells, oxidize their redox enzymes, and transfer the electrons to oxygen. Enteric bacteria protect themselves from these compounds by activating the SoxRS regulon (52, 53). Its components elevate the titer of cytoplasmic SOD, pump out the drugs, and modify the cell envelope to diminish their entry (54).

Over the past dozen years microbiologists have examined the possibility that other bacterial stresses might also owe their potency, in part, to oxidative stress. The clinical antibiotics ampicillin, kanamycin, norfloxacin and trimethoprim have been particular foci of these studies, but similar hypotheses have been ventured for metal overload, nanoparticles, solvent stress, toxin/antitoxin systems, and many others (55). Key observations have been that the stressed cells accumulate oxidized forms of cell-penetrating dyes, which are thought to be oxidized by hydroxyl radicals; that cell death is slowed by the administration of thiol compounds, which might scavenge ROS, and by iron chelators that would block hydroxyl-radical formation; and that toxicity is diminished in mutants whose TCA cycle is blocked, ostensibly diminishing the rate of respiration and any associated ROS formation (56). However, this interpretation has been challenged (55, 57–59). Most of these protectants also have the capacity to slow metabolism, and it is well known that the efficacy of antibiotic action depends upon a robust growth rate. Moreover, tests of antibiotic action did not detect oxidative damage to ROS-sensitive enzymes or to DNA; the rate of H_2_O_2_ formation (measured in scavenging mutants) was not accelerated; and the *E. coli* response to H_2_O_2_ stress (below) was not triggered. Finally, no clear model has emerged to explain how such diverse stresses could create toxic levels of ROS. More work must be done to resolve these contradictory observations.

### Exogenous Sources of Hydrogen Peroxide

H_2_O_2_ is chemically formed in habitats though abiotic reactions between sulfur and oxygen at oxic/anoxic surfaces and the photochemical reduction of oxygen by chromophores (60–63). Levels can reach 1 µM in the ocean (64, 65). It can also be produced as part of the plant wound response, during the inflammatory response of mammalian hosts—and, notably, as a primary metabolic by-product of many lactic acid bacteria (66–70). The latter organisms often lack respiratory chains and use the two-electron reduction of molecular oxygen to recycle reduced NADH, thereby indirectly improving the ATP yield of what is otherwise a fermentative process. It seems likely that the excreted H_2_O_2_ may suppress the growth of competitors. The ability of lactic-acid bacteria to tolerate their own H_2_O_2_ is impressive: They can achieve high densities in lab cultures in which millimolar H_2_O_2_ has accumulated. This tolerance appears to be due to the absence of oxidant-sensitive dehydratases and mononuclear Fe(II) enzymes. This adaptation is not without a price: These bacteria are unable to synthesize many amino acids, and they lack a TCA cycle and the improved energy yield that comes with it.

Unlike superoxide, H_2_O_2_ is an uncharged, albeit polar, molecule that can cross cell membranes. Because this process is relatively slow, when bacteria venture into environments containing extracellular H_2_O_2_, the high activities of intracellular catalases and peroxidases succeed at lowering internal H_2_O_2_ concentrations below that of the external environment (12). The transmembrane gradient for *E. coli* has been estimated to be 5- to 10-fold between the external world and the cytoplasm. Indeed, although 0.5 µM internal H_2_O_2_ is sufficient to impair the growth of this bacterium in lab cultures, external concentrations of up to 5 µM seem to be tolerated without an overt growth defect (60). Thus the limited permeability of membranes to H_2_O_2_ is essential to the efficacy of scavenging enzymes and to the ability of bacteria to grow in many habitats.

Of great interest to biologists is the role that phagocyte-derived H_2_O_2_ may play in suppressing microbial infection (**Figure 5**). The ${\text{O}}_{2}^{-}$ that is produced by host NADPH oxidase will dismutate, either spontaneously or *via* enzymic catalysis, to generate H_2_O_2_. The ability of H_2_O_2_ to cross membranes likely enables it to enter phagocytosed bacteria—but it also allows it to diffuse across the phagosomal membrane, into the producing cell, and potentially out into extracellular environments. Modeling suggests that this effusion sharply limits the amount of H_2_O_2_ inside the macrophage phagosome, despite the rapid rate at which it is formed. Estimates are that the steady-state level falls well below 10 µM (46). Such doses may be enough to induce stress responses in the captive bacteria, but they are unlikely to be lethal. The major caveat to this analysis is that it presumes that the environment acts as a one-way sink for the H_2_O_2_. However, if H_2_O_2_ accumulates within inflamed tissue, the H_2_O_2_ flow is bidirectional, and the level that accumulates may in principle be far higher. Clearly, this question cries out for direct measurements of H_2_O_2_ *in vivo*.

The rate of H_2_O_2_ production in neutrophils is substantially higher than that in macrophages—but the fact that H_2_O_2_ is a substrate of myeloperoxidase has once again been projected to cap the level at which it can accumulate (47). In sum, although at first blush it seems a no-brainer that H_2_O_2_ would contribute mightily to the killing actions of these cells, that point is not yet resolved.

## The Role of OxyR During Hydrogen Peroxide Stress

When external levels of H_2_O_2_ exceed a few micromolar, its flux into microbes threatens to elevate its internal concentration to toxic levels, despite the action of scavenging enzymes. To cope, virtually all microbes possess inducible stress responses that are focused upon H_2_O_2_. The paradigmatic system is the OxyR response of *E. coli* (71). OxyR is a H_2_O_2_-activated transcription factor. It is not activated merely by movement of *E. coli* into oxic environments, and *oxyR* mutants are capable of normal aerobic growth. However, OxyR is activated when exogenous H_2_O_2_ accumulates to ~ 0.2 μM in the cytoplasm, which is achieved by about 3 µM external H_2_O_2_ (60, 72). Null mutants cannot grow at these levels of environmental H_2_O_2_.

**Figure 6:**
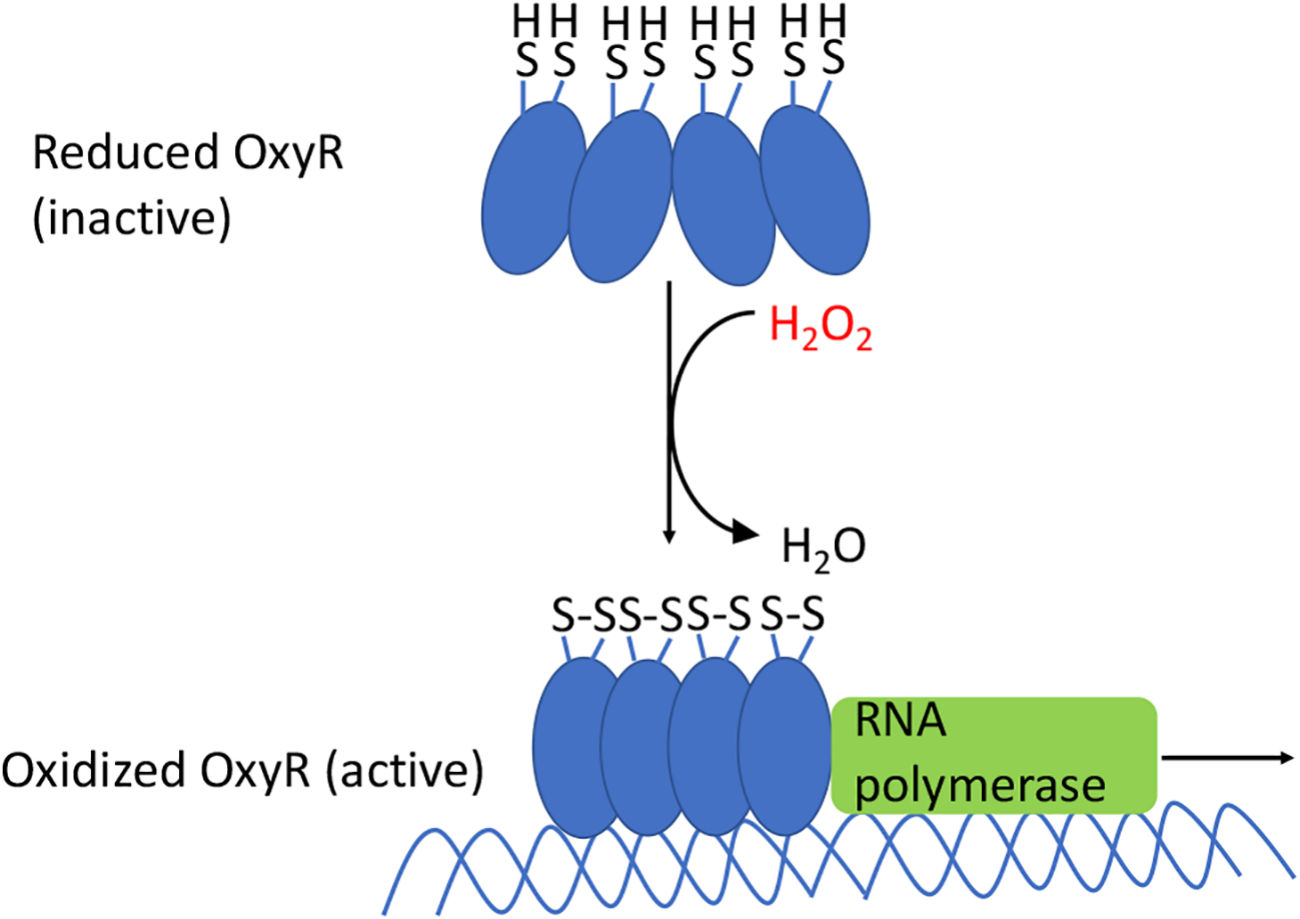
OxyR activation in *E. coli*. The oxidation of the sensory C199 cysteine by H_2_O_2_ leads to the formation of a disulfide bond between C199 and C208. The resulting conformational change causes OxyR to bind as a tetramer to the promoter regions, which recruits RNA polymerase, and results in the transcription of genes in the OxyR regulon. In many other bacteria the reduced form also binds DNA, albeit in an elongated conformation that represses transcription; oxidation again converts it to a transcriptional activator.

The OxyR protein contains a sensory cysteine residue (C199) that is directly oxidized by H_2_O_2_, generating a sulfenic acid (-SOH) (73). As a result, the residue moves from the hydrophobic pocket in which it is buried and swings toward the C208 residue, which then condenses to form a disulfide bond (**Figure 6**). This bond locks OxyR into its activated conformer, and its DNA binding ability differs from that of the reduced protein. In *E. coli* the reduced form has little transcriptional impact upon most genes, but the oxidized form recruits RNA polymerase and thereby activates the expression of genes that possess an OxyR binding site. In many other bacteria, reduced OxyR acts as a repressor, and upon its oxidation it releases the DNA, stimulating gene expression (74, 75). In still other bacteria gene expression is both repressed by the reduced form and activated by the oxidized form. The conformational change is manifested by an alteration of its DNA footprint (76).

Free cysteine reacts very slowly with H_2_O_2_ (2 M^-1^ s^-1^), and typical cysteine residues of proteins do, too (77). Yet in OxyR the sensing cysteine residue reacts with a rate constant of 10^5^ allowing it to detect micromolar H_2_O_2_ in seconds (72, 78). In that respect the hyperreactive cysteine of OxyR resembles the catalytic cysteine residue of thiol-based peroxidases, including AhpC of *E. coli.* In the latter enzyme an adjacent cationic residue facilitates the deprotonation of cysteine, which provides an order-of-magnitude improvement in its reactivity with H_2_O_2_ (79). A plausible explanation for the remaining enhancement is that the nucleophilic cysteine is arranged in a large hydrophobic cleft. Surrounding hydrogen bonds could polarize the dioxygen bond, making it vulnerable to attack, and one of the residues can protonate the hydroxide leaving group, pulling the reaction forward. As a result, thiol-based peroxidases have a rate constant of 10^7^M^-1^ s^-1^, which is appropriate for their physiological role (80, 81). A similar physical arrangement may explain the high rate constant of OxyR as well.

It follows that the activation of OxyR is an excellent marker of H_2_O_2_ stress. This effect can be tracked by monitoring the expression of OxyR-controlled genes—or by visualizing the intrinsic fluorescence of HyPer, an engineered chimera of OxyR and yellow-fluorescent protein (82). HyPer is as responsive to H_2_O_2_ as is OxyR itself, and its oxidation status can be visualized either by microscopy or flow cytometry. Importantly, two-wavelength analysis can correct for variable HyPer content in different samples, thereby avoiding loading artifacts that can arise when redox-active dyes are employed as ROS sensors.

## The Defenses That OxyR Turns on

When *E. coli* is stressed by an influx of H_2_O_2_, activated OxyR stimulates the transcription of over two dozen genes (83). These mainly fall into three categories: proteins that reduce the H_2_O_2_ concentration, proteins that shrink the iron pool, and proteins that deal with the damage that H_2_O_2_ produces (**Table 1**). The peroxidase AhpCF and the catalase KatG are each induced more than 10-fold in order to scavenge H_2_O_2_ (**Figure 2**). Why two enzymes? AhpCF is an efficient scavenger when the H_2_O_2_ concentration is less than 10 μM and the cell is well-fed (10). However, AhpCF requires NADH as a reductant, and so its activity becomes limited when catabolic substrates are scarce. In contrast, catalases do not require reductants, and they can degrade H_2_O_2_ faster than Ahp. However, catalases are problematic when the H_2_O_2_ concentrations are low, since their two-step catalytic cycle can stall with the heme in its intermediate ferryl radical form. This species is a potent oxidant, and unless it is quenched by a reductant, it can abstract electrons from the surrounding polypeptide and inactivate the enzyme (81). The KatG catalase of *E. coli*—and of many other bacteria—has a channel that apparently enables small-molecule reductants to approach the active site and quench the high-valence heme (84)**;** for this reason the enzyme is denoted a catalase/peroxidase, though the peroxidase activity by itself is too slow to comprise an efficient scavenging mechanism (85).

**Table 1:** Genes induced by OxyR during hydrogen peroxide stress in *E. coli*.

Gene	Function	Role during H_2_O_2_ stress
*ahpCF*	NADH peroxidase	Scavenge H_2_O_2_
*katG*	Catalase	
*ccp*	Cytochrome c peroxidase	Uses H_2_O_2_ as a terminal electron acceptor
*dps*	Mini-ferritin	Reduce the intracellular iron pool
*fur*	Repressor of iron import	
*yaaA*	Unknown	
*clpSA*	Chaperone	Activate Fe/S enzymes
*sufA-E*	Iron sulfur-assembly
*hemF*	Coproporphyrinogen III oxidase	Heme synthesis
*hemH*	Ferrochelatase	
*mntH*	Manganese importer	Activate mononuclear Fe enzymes
*gor*	Glutathione reductase	Maintain the thiol status
*trxC*	Thioredoxin	
*grxA*	Glutaredoxin 1	
*oxyS*	Non-coding RNA	Role unknown
*isrC*	Non-coding RNA	
*flu*	Antigen 43	
*fhuF*	Ferric iron reductase	

**Figure 7:**
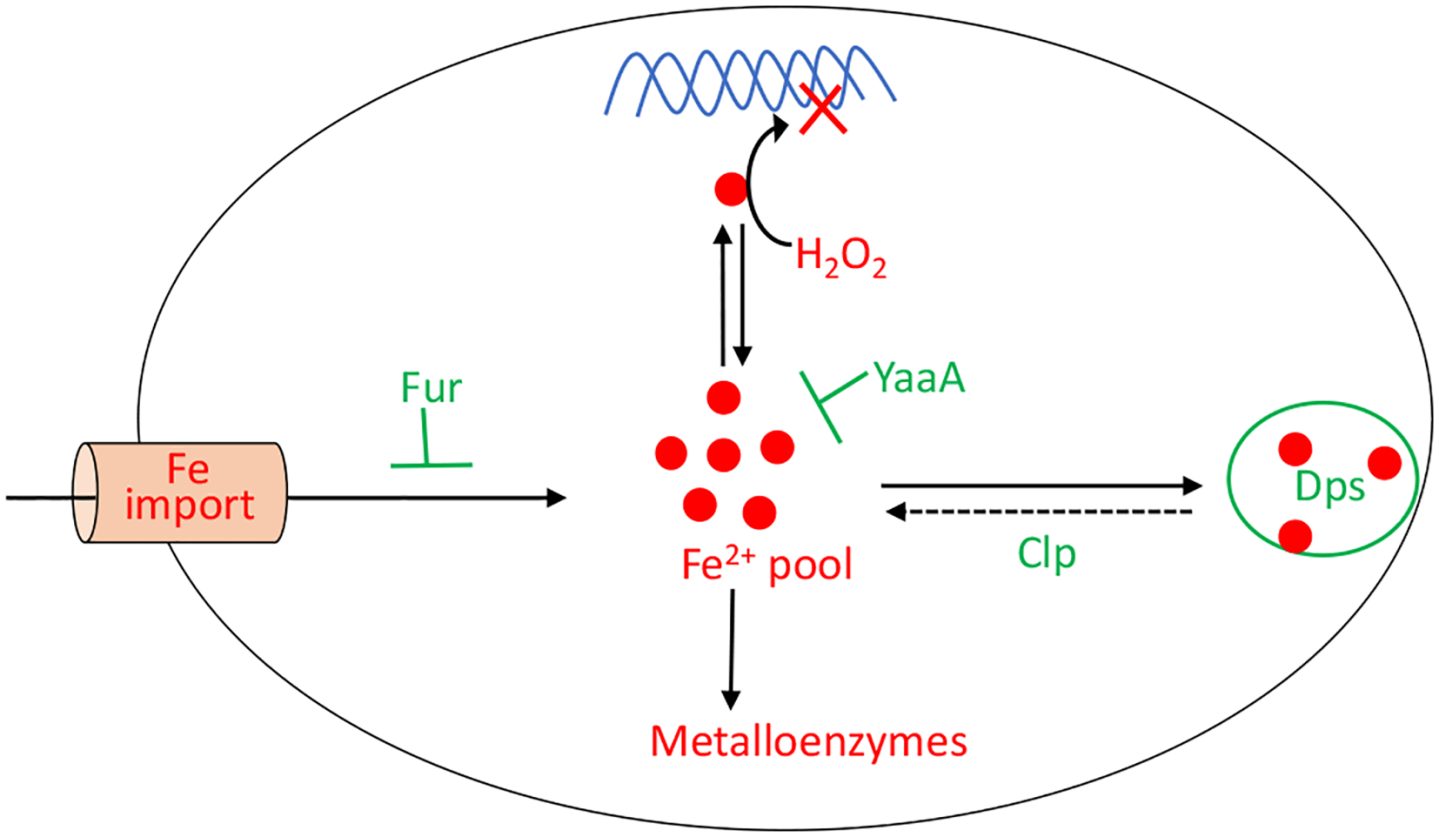
OxyR control of the intracellular iron pool. In order to minimize DNA damage, OxyR decreases the intracellular iron pool by inducing Dps, YaaA, and Fur. The Clp system maintains a small residual iron pool to enable synthesis of iron-dependent enzymes. The H_2_O_2_-responsive PerR regulon in *Bacillus subtilis* also controls Fur and MrgA, which is a Dps homolog.

When H_2_O_2_ levels are high, the existential threat to bacteria is that DNA oxidation will prove lethal. This damage is driven by the intracellular iron pool (86), and so the OxyR system employs several mechanisms to diminish it (**Figure 7**). Dps, a dodecameric mini-ferritin, is induced to sequester unincorporated iron in its hollow core (87–89). This action requires that loose Fe(II) be oxidized to Fe(III), and for this reaction Dps apparently uses H_2_O_2_ as a co-substrate. One benefit is that Dps will stop storing iron when H_2_O_2_ concentrations drop. The iron-uptake repressor Fur is also induced (90). In unstressed cells Fur:Fe(II) complexes signal that the cell has sufficient iron, and this form occludes the promoters of genes that encode iron import systems (2). When H_2_O_2_ is present, the oxidation of Fe(II) by H_2_O_2_ can deactivate Fur, potentially leading to the disastrous import of more iron. The induction by OxyR of higher levels of Fur seems to partially correct this problem (91). Finally, YaaA is a protein whose biochemical action is not understood but which demonstrably shrinks the iron pool (92). Mutants that lack any of these proteins—Dps, Fur, or YaaA—exhibit high levels of intracellular iron and suffer rapid DNA damage during protracted H_2_O_2_ stress.

While the drop in iron pools helps protect DNA, it creates a problem for the synthesis or repair of iron-cofactored enzymes. Iron cofactors comprise three main types—iron-sulfur clusters, mononuclear Fe(II) groups, and heme—and the OxyR system turns on adaptations to sustain the function of enzymes that use each. *E. coli* typically uses Isc-based machinery to build iron-sulfur clusters (93, 94), but during H_2_O_2_ stress the secondary Suf system is induced (95, 96) (**Figure 4**). For unknown reasons, this system works well when iron levels are low (97), making it better than the house-keeping Isc system, which is poisoned by H_2_O_2_ (21). The induction of a manganese importer (MntH) (98) enables mononuclear enzymes to become metallated by Mn(II) rather than Fe(II) (**Figure 3**). Manganese is not as efficient a catalyst as iron, but it remains available even as Dps sequesters iron, and it is unreactive with H_2_O_2_ and thereby enables the mononuclear enzymes to remain functional (13). Finally, continued heme synthesis is facilitated by the induction of HemH, which encodes ferrochelatase (99). This enzyme inserts ferrous iron into porphyrins to complete heme synthesis, but it could potentially become a bottleneck when Fe(II) levels are diminished. Induction of the enzyme helps to circumvent that problem.

Thus, maintaining a balance in the intracellular iron pool during H_2_O_2_ stress is challenging. The cells need to keep the levels low enough to avoid DNA damage, but not so low that the synthesis of Fe-dependent enzymes is inhibited. As one final gambit, *E. coli* uses the Clp protease system to maintain this delicate balance (27). The Clp proteins help to release some iron from Dps, allowing for the repair of [4Fe-4S] clusters (**Figure 7**). Interestingly, genetic data raise the possibility that the Clp proteins do so not by degrading Dps but perhaps by unfolding it.

Not all the members of the OxyR regulon have been explained. Glutaredoxin 1 is induced. This dithiol compound is capable of reducing protein disulfide bonds, and it helps to deactivate OxyR once the H_2_O_2_ stress has dissipated (75). However, the reasons for the induction of glutathione reductase and thioredoxin 2, which also reduce disulfide bonds, are less clear. It would be surprising if low-micromolar H_2_O_2_ directly oxidized typical protein thiols—the rate constants for these reactions are too low (37, 77). An alternative is that these systems repair [4Fe-4S] enzymes and mononuclear Fe enzymes: Cluster reactivation requires a dithiol *in vitro* (21), and the reactivation of mononuclear enzymes can require the reduction of an active-site disulfide (13). An alternative—described below—is that these sulfur reducing systems are useful if OxyR moonlights as a sensor of other thiol-derivatizing stresses.

In *E. coli* both the reduced and oxidized forms of OxyR repress *oxyR* itself by binding over its promoter; this action ensures that the titers of OxyR are controlled and do not change during H_2_O_2_ stress (76). Transcriptomic data suggest that oxidized OxyR may repress several additional genes, including those that encode the periplasmic disulfide bond chaperone DsbG, the ferric iron reductase FhuF, the inner membrane protein of unknown function YbjC, and the NADPH nitroreductase NfsA (83, 99). The significance of this regulation remains unknown.

Most members of the regulon have functions that either prevent injuries or allow the cell to tolerate them. The regulon is very successful at this: Whereas one micromolar of H_2_O_2_ in the environment fully blocks the growth of an OxyR-deficient strain, a wild-type strain is able to adapt and grow in 10 micromolar or more (60). Indeed, a final member of the OxyR regulon, cytochrome c peroxidase, is a periplasm-facing membrane-bound enzyme that allows *E. coli* to actually exploit environmental H_2_O_2_ as a respiratory oxidant when oxygen and nitrate are unavailable (100) (**Figure 2**). It is likely that the enzyme plays a role at oxic/anoxic interfaces near the intestinal epithelium, where H_2_O_2_ may be formed and may diffuse into anoxic zones.

## OxyR Is Modified Depending on the Organism

The OxyR system has been most fully studied in *E. coli*, where it is presumably adapted for the enteric environment. Similarly, other bacteria seem to have adapted the OxyR regulon to suit their particular niches; they exhibit differences in terms of the regulation mechanism, the number of OxyR homologs, and the identity of the genes in the regulon. *Porphyromonas gingivalis*, for example, encounters two types of environments: the oral cavity where the oxygen tension is high and the hemin concentrations are low, and the periodontal pockets, which contain mixed microbial communities that lower the oxygen levels and are bathed with proteins such as hemoglobin that serve as a source of hemin. Perhaps unsurprisingly, the OxyR protein in *P. gingivalis* senses both signals, as evidenced by the further activation of OxyR-regulated genes in a hemin-limited environment under anaerobic conditions (101). Interestingly, measurements of the OxyR regulon genes indicate that they are constitutively expressed. This observation is consistent with the idea that the OxyR protein has mutated into a locked-on form, presumably as an adaptation to their environment, which contains H_2_O_2_-generating lactic acid bacteria.

With a few exceptions (83), OxyR in *E. coli* predominantly acts as an activator. However, in a variety of other organisms, it acts as a repressor and/or an activator, sometimes of the same gene: Reduced OxyR is a repressor and oxidized OxyR is an activator for the catalases *katG* in *Burkholderia pseudomallei, katB* in *Shewanella oneidensis, kat* in *Neisseria meningitidis* and *Neisseria gonorrhoeae, cat* in *Corynebacterium diphtheriae*, and *katA* in *Pseudomonas aeruginosa* (102–108). In these organisms, *oxyR* mutants exhibit a higher basal level of catalase expression compared to wild-type cells, as measured by gene expression and protein measurements. The presence of H_2_O_2_ further increases these levels in a wild-type cell but not in an *oxyR* mutant, indicating both repressor and activator function. It is unclear why there is value in having OxyR act as a repressor in some organisms and as an activator in others (**Table 2**). Its action as both a repressor and an activator may create a step-function-like turn-on switch, as a modest amount of H_2_O_2_ stress may be inadequate to fully convert the OxyR population to an activator form, and the residual reduced enzyme may block the action of a subpopulation of oxidized protein. In organisms that are exposed to low, continuous levels of H_2_O_2_ stress, AhpCF may suffice to protect the cell from H_2_O_2_, but when H_2_O_2_ levels become high, a full commitment to catalase synthesis may be called for. Conversely, in other organisms it may be more beneficial to preemptively synthesize basal levels of catalase to guard against a sudden deluge of oxidative stress. Under those conditions, the basal expression can help with the initial stress, and the activation of OxyR can further increase the scavenging enzyme titers.

**Table 2:** Mechanism of OxyR control in different bacteria.

Bacteria	Oxygen tolerance	OxyR control
*Acinetobacter baumannii*	Aerobe	Activator and repressor (109)
*Caulobacter crescentus*	Aerobe	Activator (110)
*Burkholderia psuedomallei*	Aerobe	Activator and repressor (104)
*Corynebacterium diphtheriae* *Corynebacterium glutamicum*	Aerobe	Repressor (107, 111, 112)
*Deinococcus radiodurans*	Aerobe	Activator and repressor (113, 114)
*Pseudomonas aeruginosa*	Aerobe	Activator and repressor (102, 108, 115)
*Neisseria gonorrhoeae* *Neisseria meningitidis*	Aerobe	Repressor (106) Activator and repressor (103)
*Streptomyces coelicolor*	Aerobe	Activator (116)
*E. coli*	Facultative anaerobe	Activator and repressor (83)
*Haemophilus influenzae*	Facultative anaerobe	Activator (117, 118)
*Klebsiella pneumoniae*	Facultative anaerobe	Activator (119)
*Rhodobacter sphaeroides*	Facultative anaerobe	Activator (120)
*Magnetospirillum gryphiswaldense*	Facultative anaerobe	Activator (121)
*Salmonella typhimurium*	Facultative anaerobe	Activator (71)
*Serratia marcescens*	Facultative anaerobe	Activator (122)
*Shewanella oneidensis*	Facultative anaerobe	Activator and repressor (105)
*Vibrio cholerae* *Vibrio vulnificus*	Facultative anaerobe	Activator (123–125)
*Bacteroides fragilis* *Bacteroides thetaiotaomicron*	Anaerobe	Activator (126–128)
*Porphyromonas gingivalis*	Anaerobe	Activator (101)
*Tannerella forsythia*	Anaerobe	Activator (129)

Organisms such as *Vibrio cholerae, Vibrio vulnificus*, and *Deinococcus radiodurans* each deploy two OxyR proteins. In *V. vulnificus*, a facultative anaerobe that occasionally encounters aeration, the two proteins are calibrated to sense different levels of H_2_O_2_ (123–125). The more sensitive OxyR (VvOxyR2) is activated by endogenous H_2_O_2_ that is formed when the cell is aerated, whereas the less sensitive OxyR (VvOxyR1) is only activated by an influx of exogenous H_2_O_2_ from the environment. Accordingly, VvOxyR2 induces a peroxidase (VvPrx2) that has a higher activity at lower H_2_O_2_ levels compared to a second peroxidase (VvPrx1) that is induced by VvOxyR1. VvPrx1 becomes necessary because high levels of H_2_O_2_ can irreversibly over-oxidize the catalytic cysteine residue of VvPrx2. It is unclear why there are two OxyR proteins in *D. radiodurans*. The two proteins seem to regulate different genes: OxyR1 activates *katE* and represses *mntH* and *dps*, and OxyR2 represses *katG* and the hemin transport genes (113, 114).

In some bacteria the OxyR regulon includes genes that are unrelated to iron control or H_2_O_2_ degradation. Superoxide dismutase is regulated by OxyR in *P. gingivalis* and *Pseudomonas aeruginosa*, seemingly implying that superoxide stress occurs concomitant with H_2_O_2_ stress (101, 130, 131). Perhaps the obligate anaerobe *P. gingivalis*, like *Bacteroides thetaiotaomicron* (132, 133), generates toxic doses of both superoxide and H_2_O_2_ whenever it enters oxic environments; thus, it may use H_2_O_2_ as a proxy to detect aerated habitats. In contrast, *P. aeruginosa* is an aerobe—but its special feature is that in competitive environments it synthesizes pyocyanin, a redox-active compound which can produce both ROS, perhaps to poison competitors (134). This behavior may necessitate the simultaneous synthesis of both superoxide and H_2_O_2_ defenses lest *P. aeruginosa* also poison itself. Meanwhile, DNA-binding assays and transcriptional analyses have shown that in *Magnetospirillum gryphiswaldense* OxyR induces the synthesis of genes involved in magnetosome production (121). Magnetotactic bacteria make internal magnetic particles as a way to orient themselves and dive into deeper water where there is less oxygen to impair these oxygen-sensitive bacteria; one infers that H_2_O_2_ is an environmental signal that triggers this defensive taxis. Together, these data indicate that OxyR has been adapted by different organisms in ways that fit their unique environmental niche.

A key question is whether OxyR plays a leading role in defending bacteria from the H_2_O_2_ that phagocytes produce as part of the cell-based immune response. Calculations predict phagosomal H_2_O_2_ levels (47, 66) that are adequate to activate OxyR, at least in *E. coli* (60), and local H_2_O_2_ levels could conceivably rise far higher in contained environments, such as abscesses. Infection data support this idea. OxyR is important in the colonizing ability of pathogens such as *E. coli* O1:K1:H7, *Bacteroides fragilis*, and *Hemophilus influenzae*: Mutants lacking *oxyR* were unable to colonize animal models or to induce abscesses in competition assays where they were mixed with wild-type cells (118, 126, 135). Biofilms can also shelter bacteria from external stressors—perhaps including H_2_O_2_ that is produced by an inflammatory response. The OxyR responses of *Serratia marcescens*, *Neiserria gonorrhoeae, Klebsiella pneumoniae*, and *Tannerella forsythia* mediate biofilm formation, a process that helps the bacteria to persist in hosts (119, 122, 129, 136). The OxyR system is required for swarming motility and the production of exotoxins by *P. aeruginosa*: *oxyR* mutants were non-motile on plates and were unable to inhibit dendritic cell proliferation (134, 137). However, while these observations demonstrate a role for OxyR in coping with host environments, they do not directly implicate the host response as the source of the H_2_O_2_ stress. Indeed, models of urinary tract infection demonstrated that *oxyR* mutants of *E. coli* were unsuccessful at colonization—but this phenotype persisted in a host that lacked its phagocytic NADPH oxidase (135). Presumably growth of the mutant was inhibited by H_2_O_2_ that was created by other environmental sources, such as competing lactic acid bacteria.

## The Thiol-Sensing Mechanism of Yap1p

**Figure 8:**
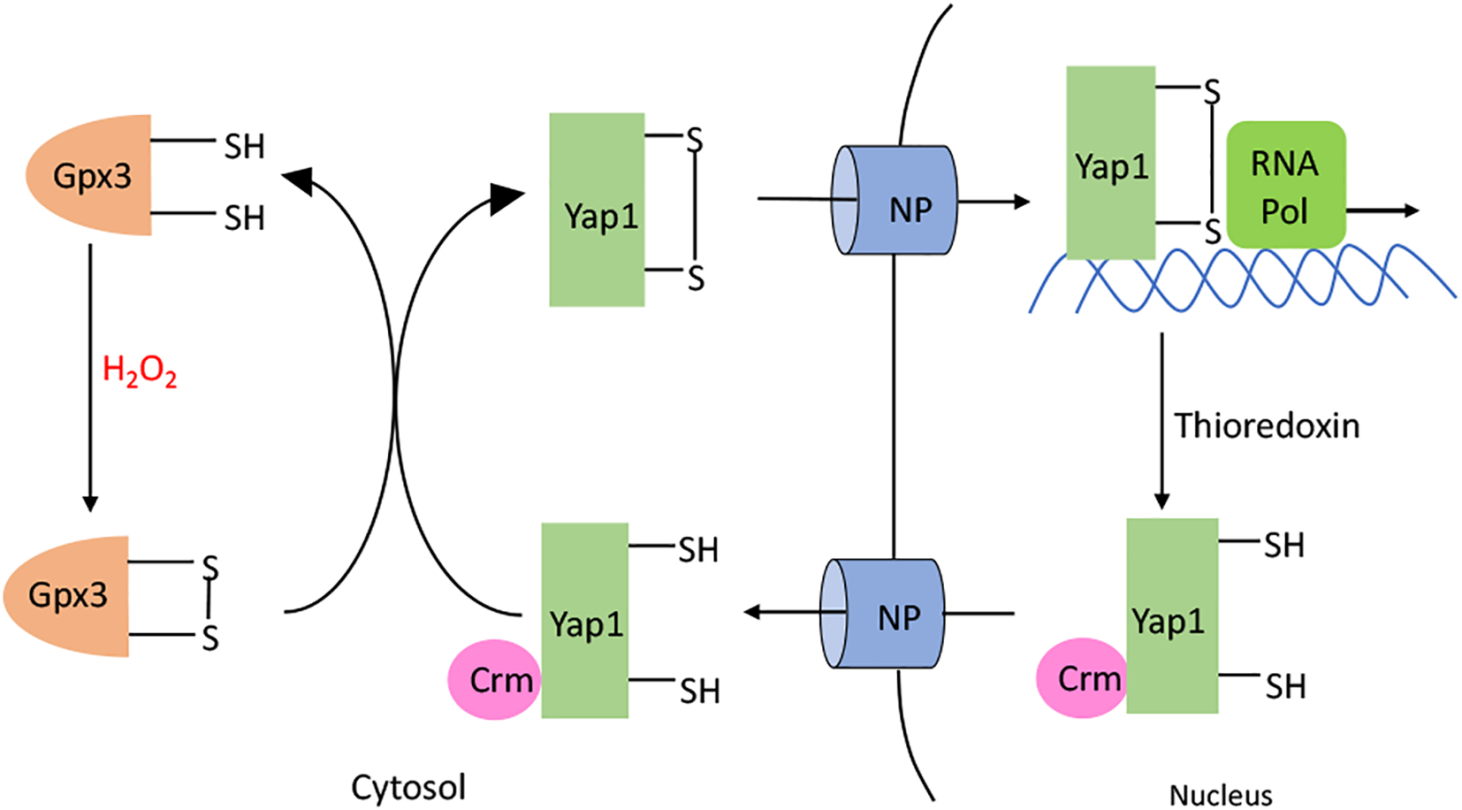
Yap1p activation. H_2_O_2_ oxidizes the C36 residue of glutathione peroxidase (Gpx3). The resulting sulfenate interacts with C598 in the C-terminal domain of Yap1p to form a intermolecular disulfide bond. Subsequent thiol-disulfide exchange reactions produce C303-C598 and C310-C629 disulfide bonds in Yap1p. Yap1p accumulates in the nucleus, leading to the activation of the Yap1p regulated genes. When H_2_O_2_ diminishes, Yap1p is reduced by the thioredoxin system; this change makes its nuclear export signal accessible to Crm, causing Yap1p to be transported back out of the nucleus.

The model eukaryote *Saccharomyces cerevisiae* also activates a defensive response when it senses hazardous H_2_O_2_ in its environment—but although it depends upon a thiol-based sensor to do so, the sensor is unrelated to OxyR. Yap1p is the key transcription factor (138). Its amino-terminus has a bZip DNA-binding domain, but in the absence of H_2_O_2_, Yap1p shuttles between the nucleus and the cytoplasm (**Figure 8**). However, when H_2_O_2_ levels rise, Yap1p is indirectly activated. The cytoplasmic Gpx3 is a glutathione peroxidase whose catalytic Cys36 residue is alternatively oxidized to a sulfenic acid by H_2_O_2_ and reduced to the thiol by glutathione, with the average redox state dictated by the level of H_2_O_2_. This sulfenic acid form can react with the C598 residue of Yap1p, which is part of the cysteine-rich domain in the N-terminus, to form an interprotein disulfide intermediate. Subsequent thiol-disulfide exchange reactions lead to the formation of an intramolecular disulfide bond between Yap1 C303 and C598, causing global conformational change. As a result, the Yap1 nuclear export signal is hidden, which blocks its interaction with the nuclear exporter Crm1 (139–141). The resultant nuclear localization of Yap1p results in the activation of several genes. Thus, unlike OxyR, the cysteine residues of Yap1p do not directly react with H_2_O_2_. This difference may ensure that Yap1p stays activated even after migrating into the nucleus, where the H_2_O_2_ may not be as high as in the cytoplasm.

In broad outline, the membership of the Yap1p regulon overlaps with that of the OxyR regulon (**Table 3**). These proteins scavenge H_2_O_2_, change iron levels, and influence the thiol status of the cell (146, 147). Yap1p induces several peroxidases, including Ahp1, Gpx2, and Tsa1, that scavenge cytosolic H_2_O_2_; it is currently unclear why there are three such systems. The glutathione reductase *GLR1* is induced to reduce the glutathione disulfide that is formed when Gpx2 reduces H_2_O_2_. Additionally, Yap1p also drives synthesis of Ctt1, a cytosolic catalase. The mitochondrial iron exporter Mmt1 is also induced. It has been hypothesized that iron is exported in order to avoid H_2_O_2_ damage to the mitochondrial DNA (145). Alternatively, the flow of iron into the cytosol may help the repair of Fe-S clusters of H_2_O_2_-sensitive enzymes, such as LeuCD, that are localized there. Similar to OxyR, Yap1p is ultimately turned off by a thioredoxin system that consists of thioredoxins Trx1 and Trx2 and thioredoxin reductase Trr1 (148).

**Table 3:** Genes induced by Yap1p during hydrogen peroxide stress in yeast.

Gene	Function	Role during H_2_O_2_ stress
*AHP1*	Cytoplasmic alkyl hydroperoxidase	Scavenge H_2_O_2_ (142–144)
*GPX2*	Cytoplasmic glutathione peroxidase	
*TSA1*	Cytoplasmic thioredoxin peroxidase	
*CTT1*	Cytoplasmic catalase T	
*MMT1*	Mitochondrial iron exporter	Reduce the mitochondrial iron pool (145)
*GSH1*	Cytoplasmic glutamylcysteine synthetase	
*GLR1*	Cytoplasmic glutathione reductase	Maintain the thiol status (142, 146)
*TRX2*	Cytoplasmic thioredoxin	
*TRR1*	Cytoplasmic thioredoxin reductase	

The fission yeast *Schizosaccharomyces pombe* features an interesting orthologue of the Yap1 system: Nuclear localization of the Pap1 transcription factor is accomplished when it receives a disulfide bond from thiol peroxidase [reviewed in (149)]. One intriguing feature of its regulon is that it includes not only familiar H_2_O_2_ defenses but also drug-resistance genes. This feature raises the possibility that H_2_O_2_ stress is frequently imposed upon *S. pombe* by natural antibiotics, much as the linkage of drug pumps and SOD to the SoxRS system of *E. coli* reveals that redox-cycling drugs are a natural source of superoxide stress.

## The Fe-Based Sensing Mechanism of PerR

Some bacteria rely on a thiol-independent mechanism of H_2_O_2_ sensing. Unlike OxyR and Yap1p, the transcriptional repressor PerR takes advantage of the reaction between Fe(II) and H_2_O_2_ to detect the stress. Most extensively studied in *Bacillus subtilis* (150–152), PerR is a Fur homolog containing a structural zinc site and a regulatory metal binding site. It was probably easy to evolve Fur to sense H_2_O_2_ stress: Fur:Fe(II) already reacts with H_2_O_2_, and Fur and PerR are sufficiently similar in primary sequence that genomic inspection cannot reliably distinguish the two.

**Figure 9:**
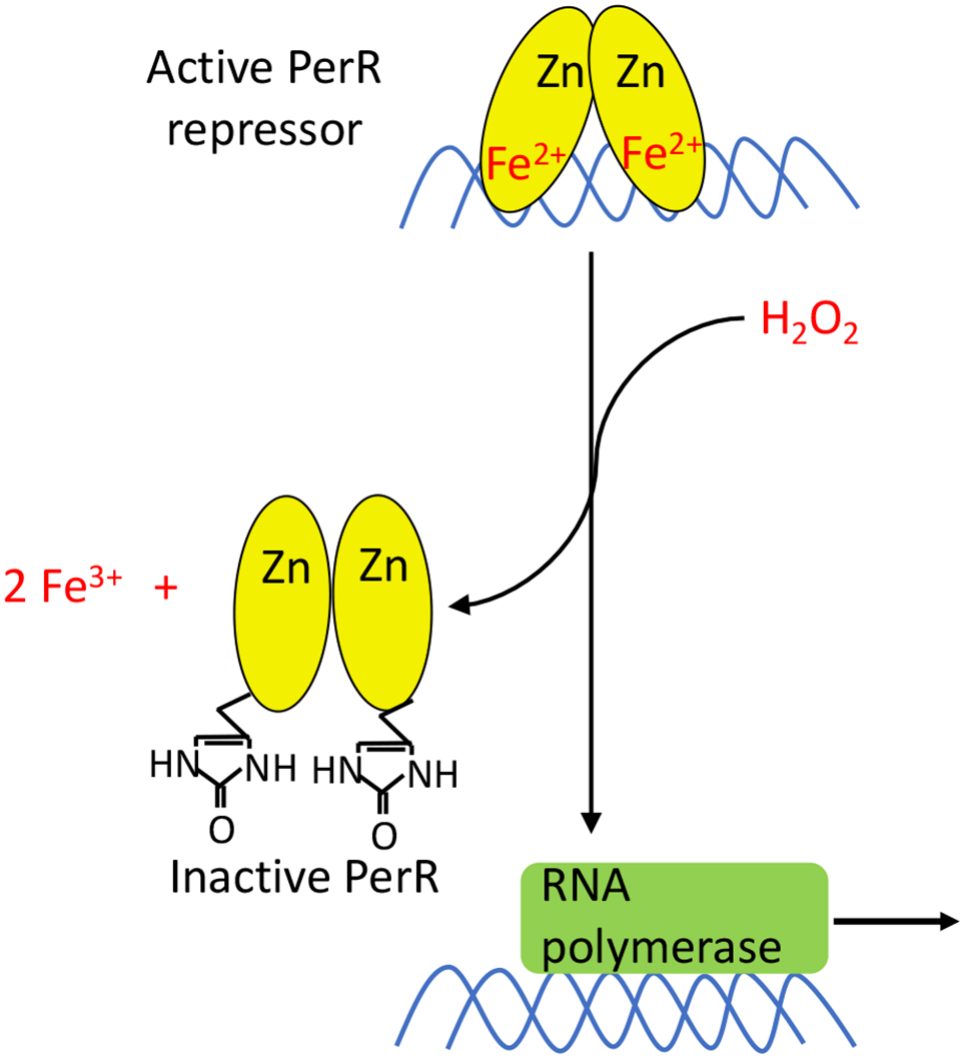
PerR activation. PerR is a dimeric DNA-binding protein, and it binds two metal ions per monomer. The first ion is a structural Zn^2+^ that is necessary for dimerization and structural integrity. The second metal ion enables DNA binding, and can either be Fe^2+^ or Mn^2+^. Only PerR bound to Fe^2+^ is responsive to H_2_O_2_. The oxidation of Fe^2+^ by H_2_O_2_ generates a localized hydroxyl/ferryl radical, which irreversibly oxidizes either of two His ligands (H37 or H91) to form 2-oxo-histidine. Metal binding is blocked, PerR dissociates from promoter sites, and the regulon is induced.

PerR acts as a dimeric repressor when it is bound to either Mn(II) or Fe(II). Under most growth conditions, PerR has a greater binding affinity for Fe^2+^. However, in iron-limited medium that has been supplemented with manganese, PerR binds to Mn(II). The identity of the metal is functionally important, as Mn-bound PerR does not react with H_2_O_2_. This is probably by evolutionary design, as manganese-rich/iron-poor cells are intrinsically less vulnerable to H_2_O_2_. Because manganese supplants iron in mononuclear enzymes and a paucity of iron precludes much DNA damage, cells need not waste resources defending themselves against H_2_O_2_. In contrast, Fe-bound PerR reacts with H_2_O_2_ (**Figure 9**) with a rate constant of 10^5^ M^-1^ s^-1^, which approximates that of OxyR (153); the similarity in rate constants suggests that the same level of H_2_O_2_ may be toxic in *B. subtilis* as in *E. coli*. The reaction oxidizes two of the His ligands bound to iron, forming 2-oxo-histidine; because the oxidized ligands cannot bind metal and the oxidation cannot be reversed, the repressor is permanently inactivated (154). Intriguingly, *in vivo* studies have shown that the majority of PerR in *Staphylococcus aureus* (PerR_SA_) is present in the oxidized form during aerobic growth, whereas this is not true of the *B. subtilis* PerR (PerR_BS_) (155). When PerR_SA_ and PerR_BS_ were alternately expressed in the same organism, the KatA activity and transcript levels of PerR-regulated genes were higher with PerR_SA_. One explanation is that PerR_SA_ is more reactive than PerR_BS_ and can sense lower concentrations of H_2_O_2_.

The inactivation of PerR results in the derepression of the PerR regulon, which again controls proteins that scavenge H_2_O_2_ and that lower the level of loose intracellular iron (156) (**Table 4**). AhpCF and KatA are induced to reduce the intracellular H_2_O_2_ levels. MrgA, which is a homolog of Dps, sequesters iron. Fur, as in *E. coli*, helps to reduce the intracellular iron levels by repressing iron import. The *hemAXCDBL* operon encodes the early steps of heme biosynthesis. Unlike *E. coli*, where OxyR induces the ferrochelatase HemH, *B. subtilis* PerR does not regulate the ferrochelatase.

**Table 4:** Genes repressed by PerR in *B. subtilis* (157, 158).

Gene	Function	Role during H_2_O_2_ stress
*ahpCF* *katA*	Alkyl hydroperoxidase Catalase	Scavenge H_2_O_2_
*mrgA* *fur*	Dps homolog Repressor of iron import	Reduce the intracellular iron pool
*hemAXCDBL*		Heme synthesis

Interestingly, the constitutive expression of the *B. subtilis* PerR regulon—in a *perR* mutant—causes trouble by excessively lowering the pool of intracellular iron (159). These mutants are more resistant to H_2_O_2_ but have trouble growing. It is unclear which enzyme-activity deficiency causes the poor growth. The iron deficiency was tracked to the combined repression of iron uptake by Fur, plus iron depletion due to the induction of KatA. Under these inducing conditions KatA becomes the single most abundant protein in the cell, comprising a whopping 10% of the total cell protein (159). This situation is reminiscent of OxyR-driven iron deficiency in *E. coli*; however, the latter is abated by induction of the Clp system (27). It seems, then, that whereas OxyR induction does not interfere with growth—and, indeed, can support it *via* the service of cytochrome c peroxidase—the induction of PerR is an emergency response that is incompatible with continued growth. Perhaps *B. subtilis* is wired to enter a period of stasis when exposed to H_2_O_2_ stress, with growth resuming only after the threat has passed, whereas OxyR allows *E. coli* to adjust and continue growing.

After H_2_O_2_ stress, the inactivated PerR is degraded by the protease LonA, and the repression of the PerR regulon is restored when the newly synthesized PerR binds either Mn or Fe (160).

## Bacteria Use PerR Differently Based on Their Niche

Similar to OxyR, PerR has also been adapted by bacteria to fit their particular niches. Differences have emerged in the types of PerR, what it senses, and the genes that it controls. Most bacteria have a single PerR regulator, such as *Staphylococcus aureus, Streptococcus pyogenes, Enterococcus faecalis*, and *Helicobacter hepaticus* (161–165). On the other hand, *Bacillus Licheniformis* has one PerR and two PerR-like proteins, both of which can sense H_2_O_2_ by histidine oxidation (166). Other bacteria contain both OxyR and PerR, including *N. gonorrhoeae, B. thetaiotaomicron*, and *D. radiodurans* (133, 167, 168); it is not yet clear why they would require both sensing systems.

The importance of PerR in different organisms may reflect the circumstances under which these bacteria experience H_2_O_2_ stress. Low-level aeration induces the PerR regulon of the anaerobe *Clostridium acetobutylicum*, perhaps due to endogenous H_2_O_2_ formation (169); as oxygen levels rise, induction of the regulon is critical for cell survival. In *Campylobacter jejuni*, a microaerophile that lacks SoxRS and OxyR homologs, the superoxide dismutase *sodB* is induced in *perR* mutants (170). These observations indicate that PerR may not be limited to defending cells against only H_2_O_2_.

Surprisingly, it has been shown that *perR* mutants of *S. aureus*, *S. pyogenes*, and Group A *Streptococcus* have lower virulence and lower intracellular survival in infected macrophages (161, 164, 171, 172), even though the derepression of the PerR regulon might be expected to induce defenses against the oxidative stress these bacteria encounter in their hosts. It is possible that the constitutive induction of the PerR regulon causes secondary growth defects similar to what is seen in *B. subtilis* (159). If so, it may explain why the *perR* mutants of these pathogens are unable to colonize their hosts. In *S. aureus* and *Staphylococcus epidermidis*, PerR represses the expression of ferritin under low-iron conditions where PerR binds to Mn, and induces it in the presence of iron, indicating that like in *B. subtilis*, PerR can regulate metal homeostasis independently of oxidative stress (161, 173).

## Do OxyR, Yap1p, and PerR Usefully Detect Other Stressors?

The reactive sensors of OxyR and PerR—a hyperreactive thiol and Fe(II), respectively—can be modified by reactive species other than H_2_O_2_, and this observation raises the question of whether these transcription factors profitably respond to these other stresses. The effectors that have been examined most closely are nitric oxide (NO) and disulfide stress.

cesses (174, 175), and it is deliberately generated at toxic levels by macrophages as part of the cell-based immune response (176). It is a radical species that can pair with the unpaired d-orbital electrons of iron; as a result, NO binds heme, exposed iron-sulfur clusters, and mononuclear iron, potentially inhibiting the enzymes that possess these cofactors (177–180). Many bacteria use NO-sensing transcription factors to control the synthesis of NO scavenging enzymes. In *E. coli*, NorR is a Fe(II)-based regulator that induces the NorVW NO reductase, while NsrR is a [2Fe-2S]-containing transcription factor whose binding by NO triggers the induction of nitric oxide dioxygenase (Hmp) (181, 182). NsrR also appears to regulate a more expansive regulon, although the roles of other members are less clear (183). The *Vibrio fischeri* NsrR regulates an alternative oxidase that is more resistant to inhibition by NO than are conventional respiratory oxidases; thus, this feature of the NsrR regulon allows this squid symbiont to sustain its respiration despite the NO that is generated by its host (184). NO has a second route of toxicity, too: Its reaction with superoxide, which is also produced by macrophages, forms peroxynitrite (ONOO^-^), a potent univalent oxidant that can penetrate into phagocytosed bacteria (185).

The Stamler group has presented evidence that OxyR also provides protection against NO stress (186, 187). Null mutants grew poorly during anaerobic respiration of nitrate, a process that might release some NO. Notably, the sensory cysteine of OxyR was nitrosylated, a modification that appeared to activate OxyR so that it induced a set of genes distinct from the conventional H_2_O_2_-driven response. The *hcp* operon was among those genes, and this group has proposed that Hcp contributes to the broader nitrosylation of cellular proteins, in a way that protects cells from nitrosative stress. The chemistry by which NO would chemically derivatize the OxyR thiol is not clear; NO is a radical species, so an oxidant, perhaps iron, needs to be involved to absorb the extra electron. Derivatization by Hcp is plausible; nitrosothiols readily react with activated cysteine residues, including that of OxyR, and this modification can perturb its behavior and has even been shown to initiate catalase synthesis—although it would seem to lack value in this situation.

However, other *in vivo* studies have elicited contradictory results. Chemostat cultures of *E. coli* that were grown anaerobically in the presence of 5 μM NO induced genes associated with NorR, NsrR, and Fur but not OxyR (188). Studies that have used even higher concentrations of NO sources, such as 1 mM acidified NaNO_2_, activated NO-detoxifying systems such as *hmpA, norV*, and *norW*, but not OxyR (189). It is possible that the identity and dose of these nitrosative stressors as well as the growth conditions contributed to the discrepancies.

The role of Yap1p in protecting yeast from nitrosative stress is also unclear. Exogenous nitrosoglutathione elicited the synthesis of superoxide dismutase and catalase, and this response depended upon Yap1p (190). However, a study that used a nitric oxide donor did not detect this effect (191). The possibility exists, then, that the effects of NO and/or nitrosothiols upon OxyR and Yap1p are adventitious. Similarly, exogenous NO can react with the Fe(II) in bacterial PerR and, by inactivating the repressor, trigger induction of its regulon (192). However, members of this regulon do not provide any obvious route to remediate this stress, and so it seems likely that effect is merely incidental to the iron-binding activity of NO.

“Disulfide stress” is a term attached to conditions that create and disseminate disulfide bonds among cellular proteins. In many studies it is imposed by exposing microbes to diamide, a manmade reagent designed to create disulfide bonds from cellular thiols (193). In some bacteria diamide elicits defensive responses that include the induction of redoxin-based disulfide-reducing systems; these regulons (194, 195) are independent of the systems that detect and suppress H_2_O_2_ stress. Diamide can activate OxyR inside *E. coli*, but very high doses are needed (75). One wonders, then, what the natural circumstances are that trigger “disulfide stress” responses. Further, because OxyR-controlled redoxins have not yet been assigned a role in defraying H_2_O_2_ stress, it is formally possible that the sensory thiol of OxyR serves a second purpose of detecting and defusing thiol-targeting electrophiles.

Thus far, the only condition under which disulfide stress is known to naturally occur in *E. coli* is during periods of rapid cystine import (196). This situation arises when sulfur-limited cells, which induce all forms of sulfur importers, encounter cystine. Gross over-import of cystine results, and disulfide-exchange reactions cause disulfide bonds to be transferred from the imported cystine to cytoplasmic proteins. OxyR is modified, and it induces its regulon. Both the thioredoxin and glutaredoxin systems that it induces act to minimize the disulfide stress. Disulfide stress can also be imposed by exposing cells to antimicrobial plant compounds such as diallyl thiosulfinate and diallyl polysulfanes; the induction of *ahpC*, *trxA*, and *trxC* results (197 **)**. It seems unlikely that *E. coli* naturally encounters these chemicals.

Similar stresses can activate Yap1p of yeast. It is directly modified by diamide—without the mediation of Gpx3—and the H_2_O_2_-sensing C303 is not involved in the resultant conformational change (198). Instead, disulfide linkages are formed between C598 and C620, C620 and C629, and C598 and C629. Glutathione reductase and thioredoxin are subsequently induced. However, broadly speaking, the activation of these systems by disulfide-generating agents—and perhaps by nitrogen species—has attracted a fair amount of attention without compelling evidence that these outcomes are not accidental.

## What’s Next?

To date, the mechanisms by which H_2_O_2_ poisons bacteria have been explored primarily in test tubes. From those studies we have learned that H_2_O_2_ stress is iron-focused, driven by reactions with enzymic iron-sulfur clusters and Fe(II) prosthetic groups and with the pool of loose iron. The high rate constants of these reactions, and the abundance of vulnerable enzymes, means that low-micromolar concentrations of H_2_O_2_ suffice to bring bacterial growth to a halt. The known defenses remediate the same injuries that have been discovered, which provides confidence that the overall picture has come into shape. In *E. coli*, the most-examined microbe, it appears that the endogenous levels of H_2_O_2_ in the fully aerated bacterium falls just short of the threshold for H_2_O_2_ toxicity—and for the induction of emergency responses. Those responses, then, evidently exist to shield the cell from external H_2_O_2_.

The next step is to figure out how this information translates to real-world environments. The broad distribution of H_2_O_2_ defenses, and in particular of inducible defenses, suggests that H_2_O_2_ stress is a pervasive phenomenon that extends, at least episodically, to most biological habitats. Yet the actual circumstances and severity of oxidative stress remain poorly understood. Plausible sources of stress range from the redox collision that occurs at oxic/anoxic interfaces to the oxidative burst of mammalian and plant defenses. We infer that different microbes may encounter this stress in different circumstances, as the defensive regulons have been modified to suit their specific situations. The rationale for these differences is not always clear; in particular, we do not yet understand why some organisms use OxyR as a sensor, why others use PerR, and why a third set employ both. Thus, if we are to fully understand oxidative stress, we will need to continue to expand these studies beyond *E. coli* and yeast, and we will need to evaluate the intensity of H_2_O_2_ stress and the role of these systems in natural habitats.

For many microbiologists, the key questions concern whether host-generated H_2_O_2_ plays a role in suppressing invasion by most bacteria—and, if it does so, by which strategies dedicated pathogens manage to circumvent this stress. It may seem intuitively obvious that phagocytic H_2_O_2_ is a potent defense, but back-of-the-envelope calculations suggest that the H_2_O_2_ levels may not rise as high as workers have sometimes assumed. Part of the difficulty here is that phagocytic action has typically been studied using as prey the same professional pathogens that have, by definition, developed ways to elude the toxicity. We endorse the idea of studying the process using the 99% of bacteria that phagocytes efficiently kill.

## Author Contributions

AS is the first author and JI is the last author. Both authors have contributed to the writing. All authors contributed to the article and approved the submitted version.

## Funding

This work was supported by grant GM49640 from the National Institutes of Health.

## Conflict of Interest

The authors declare that the research was conducted in the absence of any commercial or financial relationships that could be construed as a potential conflict of interest.
